# Comparative Phenolic Profiling and Antioxidant Activity of *Clinopodium nepeta* (L.) Kuntze Shoots Cultured In Vitro Under Different Cytokinin Treatments

**DOI:** 10.3390/molecules31132296

**Published:** 2026-07-01

**Authors:** Izabela Weremczuk-Jeżyna, Weronika Skowrońska, Agnieszka Bazylko, Izabela Grzegorczyk-Karolak

**Affiliations:** 1 Department of Biology and Pharmaceutical Botany, Medical University of Lodz, ul. Muszynskiego 1, 90-151 Lodz, Poland; izabela.grzegorczyk@umed.lodz.pl; 2 Department of Pharmaceutical Biology, Medical University of Warsaw, 02-097 Warsaw, Poland; weronika.skowronska@wum.edu.pl (W.S.); agnieszka.bazylko@wum.edu.pl (A.B.)

**Keywords:** antioxidants, chlorogenic acids, *Clinopodium nepeta*, phenolic compounds, purine cytokinins, shoot proliferation

## Abstract

*Clinopodium nepeta* (L.) Kuntze is a medicinal and aromatic species of the Lamiaceae family, rich in phenolic compounds; however, studies regarding its in vitro culture, growth regulation properties and secondary metabolism remain limited. The present study investigated the effects of three adenine-type cytokinins: 6-benzylaminopurine (BAP), meta-topolin (m-TOP), and BAP riboside (r-BAP), applied at concentrations of 0.5, 1.0, 2.0 or 4.0 mg L^−1^, on shoot proliferation, biomass accumulation, phenolic profile, and antioxidant activity in *C. nepeta* shoot cultures. The phenolic constituents of hydromethanolic shoot extracts were subjected to qualitative profiling using UHPLC–DAD–ESI–MS, while antioxidant potential was evaluated using spectrophotometric assays (DPPH, ABTS, FRAP). All tested cytokinins stimulated shoot proliferation and biomass growth compared with the control; of these, m-TOP demonstrated the most pronounced positive effect, characterized by high multiplication rate and improved shoot morphology. UHPLC–DAD–ESI–MS analysis revealed the presence of numerous caffeic acid derivatives including rosmarinic acid, chlorogenic acids, salvianolic acid derivatives, and flavonoid glycosides; their accumulation was strongly influenced by cytokinin type and concentration. Notably, rosmarinic acid, the dominant phenolic constituent in the treated shoots, reached 23.28 mg g^−1^ DW under m-TOP treatment, i.e., an approximate 20-fold increase compared with the control. The extracts from shoots cultured on cytokinin-supplemented media exhibited enhanced antioxidant activity, which correlated with increased phenolic content. These relationships were confirmed by principal component analysis (PCA). Hence, *C. nepeta* shoot cultures represent an efficient in vitro system for biomass production and phenolic compound biosynthesis, and the selection of cytokinin type is a critical factor modulating both morphogenetic and metabolic responses.

## 1. Introduction

Medicinal and aromatic plants are frequently exploited in plant biotechnology due to their capacity for synthesizing structurally diverse secondary metabolites. In recent years, plant in vitro culture systems have become promising tools for both fundamental studies on plant growth regulation and the controlled production of specialized metabolites. The development of advanced analytical techniques, particularly liquid chromatography coupled with mass spectrometry, has allowed more comprehensive analysis of metabolic responses. As such, in vitro-based experimental models have become increasingly relevant as reproducible and well-defined systems [1,2].

Among the various in vitro experimental models that have been designed to date, shoot cultures represent a particularly robust and physiologically relevant system for investigating growth regulation and secondary metabolite biosynthesis under controlled conditions. In particular, they offer high genetic and biochemical stability and accumulate specialized metabolites whose biosynthesis is tightly related to tissue differentiation [3]. In addition, it is possible to precisely manipulate their environmental and nutritional conditions, thus enabling systematic evaluation of the factors regulating morphogenesis, biomass production, and secondary metabolism. Such systems have been successfully applied based on numerous medicinal plants, including *Salvia*, *Lavandula* or *Nepeta* species [4,5,6].

One of the most critical determinants of morphogenetic responses and metabolic activity in in vitro cultures is the choice of plant growth regulator. Auxins and cytokinins are routinely employed to control organogenesis and shoot proliferation; however, they also act as key modulators of secondary metabolism [7]. Cytokinins, in particular, influence cell cycle progression, cell differentiation and the regulation of phenylpropanoid biosynthesis. Cytokinin supplementation of culture media has been shown to significantly affect the accumulation of phenolic acids and flavonoids, often in a concentration- and structure-dependent manner [4].

The most widely used adenine-type cytokinin in plant tissue culture is 6-benzylaminopurine (BAP); however, increasing attention has been directed toward its derivatives, such as hydroxyl or sugar substitutes. These compounds have been reported to reduce the physiological disorders frequently associated with prolonged BAP exposure, while also promoting shoot multiplication and biomass growth, and enhancing secondary metabolite accumulation [6,8].

*Clinopodium nepeta* (L.) Kuntze (syn. *Calamintha nepeta*), a perennial species of the Lamiaceae family, is widely distributed across the Mediterranean region, Southern Europe, and Western Asia [9,10]. The species, commonly used as a culinary herb, has a long history of ethnopharmacological use in the treatment of gastrointestinal, respiratory and neurological disorders, and in treating muscle spasms [10,11].

Contemporary studies confirm that *C. nepeta* exhibits antimicrobial, antioxidant, antimutagenic, antihyperglycemic, sedative, anticonvulsant, diuretic, and hepatoprotective effects [12,13]. These properties are primarily associated with its rich phytochemical profile, particularly essential oils and phenolic compounds. Studies have confirmed the presence of various flavonoids (e.g., acacetin, quercetin, kaempferol, apigenin, and chrysoeriol derivatives) and phenolic acids, including caffeic, chlorogenic, and rosmarinic acids [12,14]. Phenolic constituents, especially caffeic acid derivatives, have been found to demonstrate significant antioxidant and anti-inflammatory activities. They can thus offer protection against oxidative stress-related disorders, including cardiovascular and neurodegenerative diseases [15,16,17]. Consequently, taxa containing these compounds—both well-characterized members of the Lamiaceae family, such as *Salvia*, *Ocimum*, and *Mentha,* and less extensively studied genera, such as *Clinopodium*—are attracting increasing attention from researchers and biotechnologists.

While *C. nepeta* has been documented to have pharmacological potential, data on its in vitro culture, and the regulation of its secondary metabolite production by cytokinins, remain scarce. Furthermore, little is known of the effects of modern cytokinins on its shoot morphogenesis and phenolic compound accumulation. Previous data from other medicinal plant species indicate that m-TOP and r-BAP may promote shoot proliferation and phenolic metabolite accumulation more effectively than conventional BAP, while reducing the incidence of physiological disorders [6,8]. However, whether such responses occur in *C. nepeta* remains unknown. Therefore, the aim of the present study was to evaluate the influence of three cytokinins, viz. BAP, meta-topolin (m-TOP) and BAP riboside (r-BAP), applied at concentrations of 0.5, 1.0, 2.0 or 4.0 mg L^−1^, on shoot proliferation, biomass accumulation, phenolic metabolism, and antioxidant activity of *C. nepeta* shoot cultures. Hydromethanolic shoot extracts were obtained; their phenolic constituents were profiled by UHPLC-DAD-ESI-MS, and their antioxidant potential determined by FRAP, DPPH and ABTS assays. While *C. nepeta* has demonstrated antioxidant potential in preliminary studies, the antioxidant activity of the in vitro-derived biomass and its modulation by cytokinins remain unknown. Therefore, antioxidant activity was evaluated to determine whether cytokinin-induced changes in phenolic metabolism were accompanied by corresponding changes in extract bioactivity, thereby providing a functional assessment of the metabolic responses of shoots cultured under different growth regulator treatments. Our findings offer a novel insight into the metabolic responses of *C. nepeta* shoots to cytokinin treatment, and support the development of protocols for the production of standardized, polyphenol-rich plant material.

## 2. Results and Discussion

### 2.1. Effect of Cytokinins on Shoot Proliferation and Biomass Accumulation of C. nepeta

*Clinopodium nepeta* nodal explant shoot proliferation was significantly influenced by cytokinin type, cytokinin concentration, and their interaction (two-way ANOVA; Table 1). Of these, the greatest impact was demonstrated by cytokinin concentration (partial *η*^2^ = 0.413), followed by type and interaction effect.

To date, in vitro propagation of *C. nepeta* and related *Clinopodium* spp. has been conducted almost exclusively using conventional cytokinins, with BAP reported as an effective regulator of shoot induction [9,18,19,20]. However, the application of BAP is often associated with physiological disorders, particularly hyperhydricity, with a negative effect on shoot quality and large-scale propagation [21].

The *C. nepeta* nodal explants demonstrated high morphogenetic competence. All tested cytokinins induced shoot formation from nodal explants, with response frequencies ranging from 55 to 85%. The highest explant responsiveness (>80%) was observed for r-BAP at all tested concentrations, and for BAP and m-TOP at higher concentrations (Table 1). These findings are consistent with earlier reports for *Clinopodium* species, in which shoot induction frequencies typically exceeded 70% when BAP was applied [9,19,20]. In addition, r-BAP and m-TOP demonstrated similar effectiveness in promoting shoot initiation with previous studies on other Lamiaceae species [22,23].

**Figure 1:**
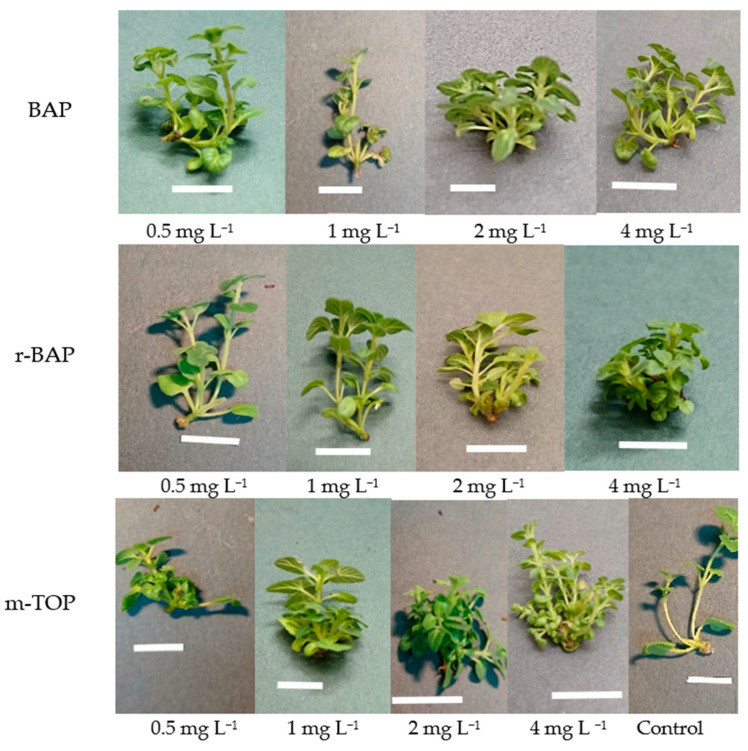
The shoot culture of *C. nepeta*. cultivated on MS medium with 0.2 mg/L IAA (C-control) and different cytokinin variants after 5 weeks. White scale bars represent 1 cm in all panels.

The results indicate a significant interaction between cytokinin type and concentration: shoot multiplication increased with concentration for all cultures, with the magnitude determined by type (Table 1). The highest number of shoots per explant was obtained on media supplemented with 4 mg L^−1^ m-TOP (4.5 shoots per explant), followed by 2 mg L^−1^ m-TOP and 4 mg L^−1^ BAP (Table 1, Figure 1). These values were markedly higher than controls (1.34 shoots per explant), confirming that exogenous cytokinins play an essential role in overcoming apical dominance and stimulating axillary bud outgrowth. The observed proliferation rates were comparable to or exceeded the optimal values previously reported for this and related species. Notably, under optimal culture conditions, the regenerated shoots exhibited superior morphology and substantially less hyperhydricity than those described in earlier studies [9,18,19,20].

Most shoots regenerated in this study were morphologically normal and well developed (Figure 1). However, hyperhydricity was noted at cytokinin concentrations exceeding 1 mg L^−1^, with pronounced differences being present among the tested regulators. The highest proportion of hyperhydric shoots (25%) was observed in the presence of 4 mg L^−1^ BAP, whereas markedly lower frequencies were recorded for m-TOP (3%) and r-BAP (11%) at the same concentration (Table 1). Hence, BAP derivatives have a clear advantage over conventional BAP in maintaining shoot quality. Similar observations have been reported for numerous species, including *Salvia bulleyana*, *Daphne mezereum*, *Aloe polyphylla*, *Pistacia* spp., and fruit tree species, where m-TOP reduced hyperhydricity while sustaining or even enhancing multiplication rates [24,25,26,27,28].

The superior performance of BAP analogues is generally attributed to their distinct metabolic fate. The presence of a ribose moiety at the N9 position, or a hydroxyl group in the meta position of the benzyl ring, enables the formation of reversible *O*-glucosides that act as a reservoir of biologically active cytokinin; they also prevent the rapid degradation of the free hormone in plant tissues and its excessive accumulation [24,29]. In contrast, BAP can be rapidly converted into inactive metabolites and, at high concentrations, may induce oxidative stress and contribute to physiological disorders [30].

While shoot elongation was significantly affected by cytokinin concentration and by the interaction between cytokinin type and concentration, cytokinin type alone did not have any significant influence (two-way ANOVA) (Table 1). The effect size of concentration was moderate (partial *η*^2^ = 0.246; *p* < 0.001), while the interaction effect was weaker but still significant (partial *η*^2^ = 0.040; *p* = 0.022). In general, shoot length decreased with increasing cytokinin concentration, with the longest shoots obtained at the lowest cytokinin levels (Table 1). This pattern is consistent with previous studies on *C. nepeta* and other Lamiaceae species: it reflects the inhibitory effect of cytokinins on shoot elongation, which is associated with reduced apical growth and stimulation of lateral bud development [9,19,31].

**Figure 2:**
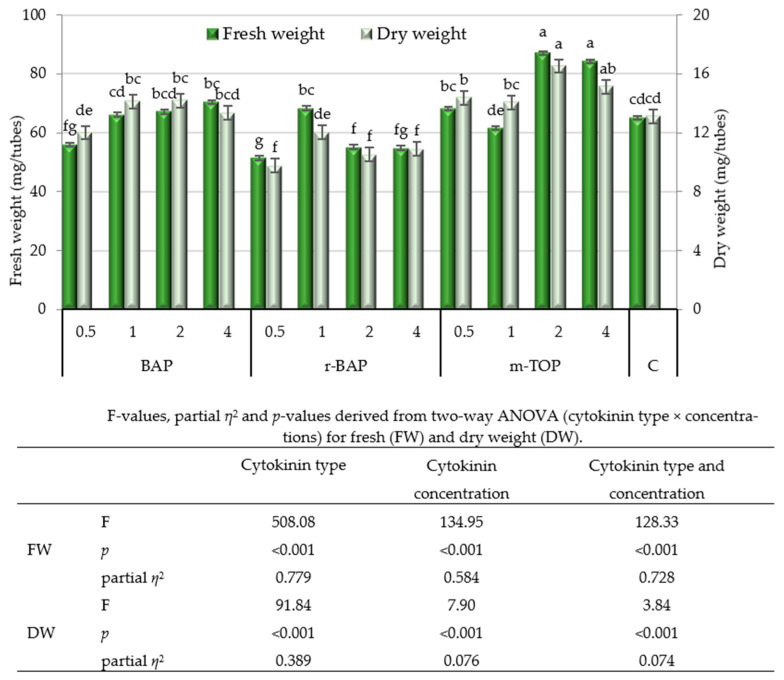
The effect of cytokinin application on *C. nepeta* culture biomass. Results expressed as means of three replicates ± SE (calculated from a two-way ANOVA model). Means followed by at least one common letter are not significantly different according to Tukey’s HSD test (*p* ≤ 0.05), with “a” indicating the highest mean value.

The tested cytokinins exhibited varying effects on *C. nepeta* biomass accumulation, a factor of particular importance in shoot culture systems intended for secondary metabolite production. Two-way ANOVA revealed that fresh biomass was significantly affected by cytokinin type and concentration, as well as their interaction (*p* < 0.001 for all factors) (Figure 2). The interaction was characterised by very high effect size (partial *η*^2^ = 0.728), comparable to that of cytokinin type, while concentration alone also had a substantial effect. Hence, biomass accumulation appears to depend largely on the specific combination of cytokinin type and concentration, rather than on either factor considered independently. Consistent with this interaction pattern, the highest fresh biomass accumulation was obtained on media supplemented with m-TOP at 2 and 4 mg L^−1^, where the fresh weight exceeded 80 mg per tube (Figure 2). These values were markedly higher than those recorded for BAP- or r-BAP-treated cultures and the control. Indeed, shoots grown in the presence of r-BAP or at low BAP concentrations exhibited the lowest fresh biomass accumulation, which in some cases was even lower than that observed in the control culture.

A similar tendency was observed for dry biomass production. Two-way ANOVA confirmed significant effect sizes for cytokinin type and concentration, and their interaction (*p* < 0.001 for all factors). However, these effects were smaller than those noted for fresh weight (Figure 2). This pattern suggests that differences in dry biomass were influenced primarily by cytokinin type, and much less by concentration alone and the interaction between the two factors.

The highest dry biomass was also recorded in cultures supplemented with m-TOP at 2 and 4 mg L^−1^, reaching 16.52 and 15.67 mg per tube, respectively (Figure 2). These values significantly exceeded those obtained in cultures treated with BAP, r-BAP or the control. The strong stimulatory effect of m-TOP on biomass production observed in *C. nepeta* is consistent with previous reports for *Salvia bulleyana*, *Stevia rebaudiana*, and *Pterocarpus marsupium*, where m-TOP promoted both fresh and dry biomass accumulation more effectively than BAP at comparable concentrations [8,28,32]. These findings indicate that m-TOP is particularly suitable for applications aimed at maximizing biomass yield in shoot culture systems.

### 2.2. Phenolic Profile of C. nepeta In Vitro Shoots Determined by UHPLC–DAD–ESI–MS

When grown under natural conditions, *C. nepeta* plant extracts are dominated by phenolic acids, particularly caffeic and rosmarinic acid derivatives, along with flavonoids and their glycosides [12,33]. However, little is known about the phenolic composition of *C. nepeta* obtained by in vitro culture.

**Figure 3:**
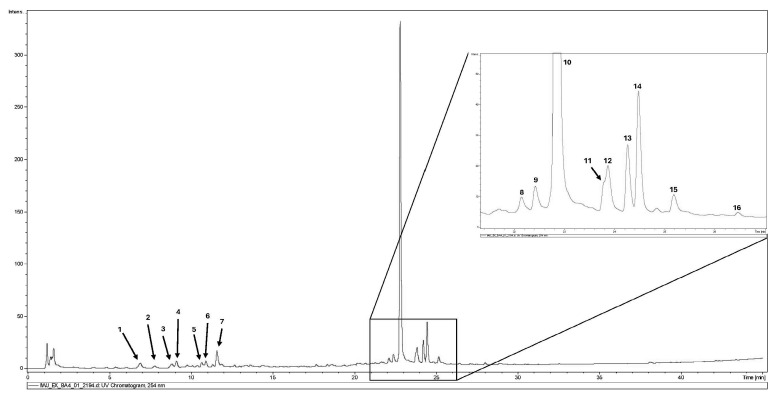
Representative chromatogram of the hydromethanolic extract of *C. nepeta* shoot culture recorded by the UPLC-DAD-ESI-MS. Peaks: 1—neochlorogenic acid, 2—dicaffeoyl glycoside, 3—caffeoyl hexoside, 4—caffeoyl-threonic acid, 5—chlorogenic acid, 6—lithospermic acid derivative, 7—cryptochlorogenic acid, 8—diosmetin rutinoside, 9—salvianolic acid D derivative, 10—rosmarinic acid, 11—clinopodic acid I, 12—lithospermic acid isomer, 13—acacetin acetylhexosyl-rutinoside, 14—acacetin 7-*O*-rutinoside, 15—salvianolic acid isomer, 16—monomethyl lithospermate isomer.

The hydromethanolic extracts were found to have a structurally diverse phenolic profile, as indicated by UHPLC–DAD–ESI–MS data. Based on accurate mass measurements, fragmentation patterns and UV–Vis spectra, and comparison with reference standards and literature data, 16 phenolic compounds, including phenolic acids and their derivatives, as well as three flavonoid glycosides, were tentatively identified (Figure 3, Table 2). Importantly, no qualitative differences in phenolic composition were observed between the tested treatments, indicating that cytokinin application primarily affected metabolite level rather than biosynthetic capability.

Three compounds (**1**, **5**, and **7**) exhibited a characteristic pseudomolecular ion [M − H]^−^ at *m*/*z* 353 (Table 2). Based on comparison of their MS fragmentation patterns with literature data [20,34,35] and chromatography with an authentic reference standard (chlorogenic acid), these metabolites were identified as caffeoylquinic acid isomers. Compound **5** was unambiguously assigned as chlorogenic acid (5-*O*-caffeoylquinic acid), whereas compounds **1** and **7** were tentatively identified as neochlorogenic acid (3-*O*-caffeoylquinic acid) and cryptochlorogenic acid (4-*O*-caffeoylquinic acid), respectively. Caffeoylquinic acids have previously been reported in the aerial parts of *C. nepeta* and other representatives of the genus *Clinopodium*, including *C. incana* and *C. vulgare*, supporting their chemotaxonomic relevance within the genus [12,20,46,47].

Compounds **2**, **3**, and **4** were tentatively characterized as caffeic acid derivatives, respectively: caffeoyl dihexoside, caffeoyl hexoside, and a caffeoyl-threonic acid isomer (Table 2). The proposed annotations were based on their fragmentation patterns, and comparison with published data [36,37]. Caffeoyl glycosides, and other derivatives, have previously been identified in *Clinopodium bolivianum* and other members of the Lamiaceae family [6,48].

Compound **10**, characterized by a pseudomolecular ion [M − H]^−^ at *m*/*z* 359, was identified as a rosmarinic acid (Table 2), a metabolite widely documented in *Clinopodium* species including *C. nepeta* [20,33,47].

Compound **12** was identified as lithospermic acid following comparison with the fragmentation pattern and retention time of an authentic lithospermic acid standard. Compound **15** was assigned as an isomer of lithospermic acid, according to previously reported spectral data [38], whereas compounds **6** and **16** displayed fragmentation patterns characteristic of lithospermic acid derivatives. In particular, the deprotonated molecular ion at *m*/*z* 551 observed for compound **16** indicated the presence of a methyl substituent, tentatively identifying it as a monomethyl lithospermic acid isomer [45]. The occurrence of lithospermic acid has previously been confirmed in *C. sericeum* [49], supporting its distribution within the genus.

All analysed samples demonstrated the presence of an additional salvianolic acid derivative (peak 9; Table 2). The compound exhibited a deprotonated molecular ion [M − H]^−^ at *m*/*z* 553, and its fragmentation pattern largely corresponded to that reported for the 2-(3,4-dihydroxyphenyl)ethyl ester of salvianolic acid D identified by Fialova et al. [40] in the genus *Mentha*. Accordingly, the metabolite was annotated as a salvianolic acid D derivative. To the best of our knowledge, this is the first report of this compound in any species of the genus *Clinopodium*.

Peak 11 displayed a pseudomolecular ion [M − H]^−^ at *m*/*z* 717 (Table 2) and was annotated as clinopodic acid I, a caffeic acid tetramer, according to published MS data [41]. To date, this compound has not been reported in *C. nepeta*; however, it has been identified in *C. chinense var. parviflorum* [50] and *C. vulgare* [47].

In addition to polyphenolic acid derivatives, flavonoid glycosides were detected in all *C. nepeta* shoot extracts. The mass spectrum of compound **8**, characterized by a pseudomolecular ion [M − H]^−^ at *m*/*z* 607 (Table 2), was consistent with literature data for a diosmetin glycoside [36,39]. Accordingly, this compound was tentatively identified as diosmetin 7-*O*-rutinoside. Although it has not previously been reported in *C. nepeta*, it has been confirmed in the *Micromeria* genus (which has close taxonomic relationships with *Clinopodium*) [51]. Compounds **13** and **14** exhibited deprotonated molecular ions at *m*/*z* 841 and 637, respectively (Table 2); these were designated as acacetin 7-*O*-rutinoside (peak 13) and acacetin acetylhexosyl-rutinoside (peak 14). Various acacetin glycosides have previously been reported in *C. nepeta* and its subspecies, as well as in other representatives of the genus [33,43].

### 2.3. Effect of Cytokinins on Phenolic Compound Accumulation

**Figure 4:**
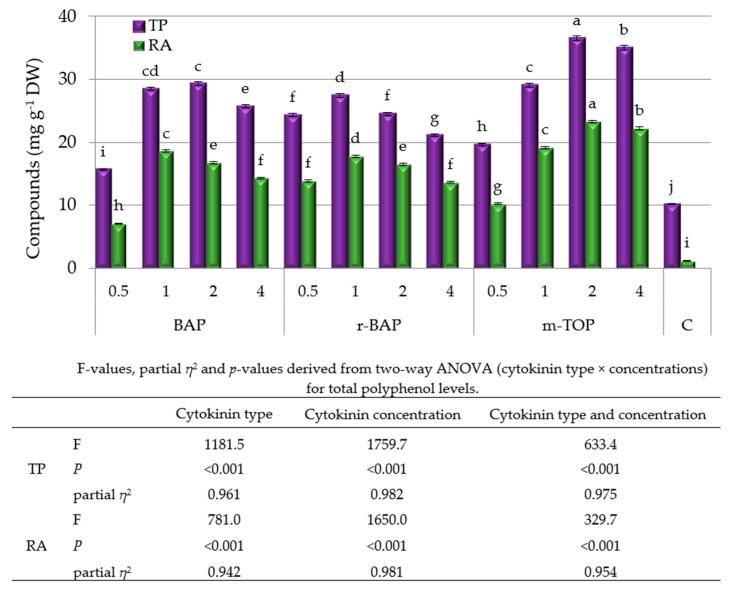
The effect of cytokinin on total polyphenols (TP) and rosmarinic acid (RA) content in *C. nepeta* culture. Results expressed as means of three replicates ± SE (calculated from a two-way ANOVA model). Means followed by at least one common letter are not significantly different according to Tukey’s HSD test (*p* ≤ 0.05), with “a” indicating the highest mean value.

Although the qualitative phenolic profiles of *C. nepeta* shoots remained largely unchanged, the quantitative accumulation of individual compounds was strongly influenced by cytokinin type, concentration, and the interaction between these two factors (two-way ANOVA; Figure 4). The highest total phenolic content, i.e., the sum of all identified phenolic metabolites quantified in the extracts, was recorded in shoots cultured on medium supplemented with 2 mg L^−1^ m-TOP, followed by medium with 4 mg L^−1^ m-TOP (36.49 and 35.00 mg g^−1^ DW, respectively); these values represent more than a threefold increase compared with control shoots (Figure 4).

The accumulation of individual analysed phenolic compounds detected in *C. nepeta* shoots was significantly influenced by cytokinin type and concentration, and the interaction between these two factors (*p* < 0.001; two-way ANOVA). For most metabolites, the interaction effect accounted for the largest proportion of variance, with partial *η*^2^ values exceeding 0.95 (Table 3, Table 4 and Table 5). The only exception was caffeoyl hexoside, for which the interaction effect was slightly lower, but remained high (partial *η*^2^ = 0.791). These results indicate that the accumulation of individual metabolites was largely determined by specific combinations of cytokinin type and concentration.

In the cytokinin-treated shoots, the dominant phenolic compound was rosmarinic acid (RA), with concentrations ranging from 7.12 to 23.28 mg g^−1^ DW. Its accumulation was markedly stimulated by m-TOP, reaching maximum levels at 2 and 4 mg L^−1^, which were nearly twenty times higher than those recorded in control cultures (Figure 4).

Considering that its content in wild plants (before the flowering stage) reached 9.7 mg g^−1^ of dry extract [52], our results suggest that optimized shoot cultures of *C. nepeta* may constitute a promising model for RA production. Moreover, it should be noted that this value will correspond to a substantially lower level once recalculated and expressed per dry plant material, depending on the extraction yield. For instance, Gonçalves et al. [46] reported RA content of 1.44 mg g^−1^ dry extract from leaves of soil-grown *C. nepeta*, with an extraction yield of 23.89%. When calculated, this corresponds to approximately 0.34 mg g^−1^ DW of plant material, i.e., over one order of magnitude lower than the concentrations obtained in the present study. Comparable trends were observed in other representatives of the genus. In *C. sylvatica*, RA levels ranged from 0.076 to 0.814 mg g^−1^ DW, which are markedly lower than those recorded here [53]. In contrast, in *C. vulgare*, RA content, determined by HPLC, reached 12.87 mg g^−1^ DW in the leaves of in vitro-derived plants and up to 17.35 mg g^−1^ DW in those of wild-growing plants [20]. These values are within the upper range of accumulation reported for the genus but remain lower than those achieved in *C. nepeta* shoots under optimized in vitro conditions.

These comparisons indicate that the treated *C. nepeta* shoot cultures are capable of accumulating high levels of RA, exceeding those reported for soil-grown plants, as well as for other *Clinopodium* species cultivated both in vitro and in vivo. Hence, this culture system offers considerable potential as a controlled source of phenolic acids for pharmaceutical use.

Other phenolic acids, including caffeoylquinic acids, were present at lower concentrations, and showed distinct accumulation patterns depending on cytokinin treatment (Table 3). The content of caffeoylquinic acids NCHA, CRCHA and CHA ranged from 0.05 to 1.21 mg g^−1^ DW. Of these, CRCHA was pre-dominated, with the highest concentrations recorded in shoots cultured in the presence of 2 and 4 mg L^−1^ m-TOP.

In comparison, substantially lower levels of these compounds were reported in soil-grown *C. nepeta*. After recalculation of the data of Gonçalves et al. [46], the contents of neochlorogenic, chlorogenic and cryptochlorogenic acids correspond to approximately 0.09, 0.07 and 0.35 mg g^−1^ DW of plant material, respectively. These values are markedly lower than or at most comparable to the concentrations obtained in the present study, particularly for CRCHA, indicating that in vitro cultured shoots of *C. nepeta* under cytokinin treatment enhance not only rosmarinic acid level but also the flux towards caffeoylquinic acid derivatives.

In comparison, substantially higher levels of caffeoylquinic acids have been reported in *C. vulgare* [20]. NCHA and CHA reached up to 5.96 and 2.06 mg g^−1^ DW, respectively, with comparable concentrations observed in leaves of wild-growing plants and in vitro-derived shoots. Hence, in this species, the accumulation of these metabolites is primarily influenced by genotype and organ type than by in vitro conditions. However, data for *C. sylvatica* indicate generally low accumulation of individual phenolic acids relative to total phenolics [53]. In contrast, the caffeoylquinic acids observed in *C. nepeta* shoots generally demonstrate relatively high levels that are influenced by cytokinin application; this further indicates that in vitro systems enable targeted modulation of the phenylpropanoid pathway, extending beyond rosmarinic acid biosynthesis.

In contrast to most quantified polyphenolic acids, CLA predominated at higher BAP concentrations (Table 4). These differences suggest that individual cytokinins may differentially regulate phenylpropanoid biosynthesis, thereby contributing to the accumulation of specific phenolic compounds. However, the underlying regulatory mechanisms remain to be elucidated.

Cytokinin supplementation also stimulated flavonoid glycoside accumulation in culture of *C. nepeta*. The highest total flavonoid content exceeded 8 mg g^−1^ DW and was observed in shoots cultured on media containing 2 and 4 mg L^−1^ m-TOP (Table 5). The predominant flavonoids were acacetin derivatives, with acacetin 7-*O*-rutinoside and acacetin acetylhexosyl-rutinoside reaching their maximum levels at 2 mg L^−1^ m-TOP (3.78 and 3.66 mg g^−1^ DW, respectively), i.e., approximately 20–25% higher than in the control shoots.

In contrast, the dominant flavonoid in soil-grown *C. nepeta* was quercetin-3-*O*-rutinoside, reported at 5.2 mg g^−1^ of dry extract, corresponding to approximately 1.24 mg g^−1^ DW of plant material, i.e., markedly lower than the concentrations of individual acacetin glycosides obtained in the present study [46].

A different flavonoid profile was observed in *C. vulgare*, where quercetin derivatives predominated. In this species, quercetin content in the stems of in vitro-derived shoots reached 5.7 mg g^−1^ DW, exceeding the values noted in wild-growing plants (3.2 mg g^−1^ DW) [20]. Hence, flavonoid accumulation in *Clinopodium* spp. appears to be species specific and differentially responsive to in vitro conditions: *C. vulgare* preferentially accumulates quercetin derivatives, while *C. nepeta* favours acacetin glycosides.

Additionally, the phenolic profile of the in vitro *C. nepeta* shoots was strongly influenced by cytokinin treatment. In many cases, higher cytokinin concentrations promoted increased accumulation of phenolic compounds, although the magnitude of this response depended on the cytokinin applied. Higher levels of metabolites were observed following m-TOP treatment compared with BAP and r-BAP.

Several plant species exhibit elevated phenolic metabolism following treatment with BAP derivatives, with the response influenced by species, cytokinin type and concentration [6,8]. For example, in *Dracocephalum forrestii* shoot cultures, m-TOP and r-BAP induced threefold and twofold higher chlorogenic acid accumulation, respectively, compared with pure BAP [22]. In *Salvia bulleyana*, m-TOP also increased the total content of phenolic acids, including caffeic and salvianolic acid derivatives [28]. Moreover, in *Stevia rebaudiana*, m-TOP effectively stimulated chlorogenic and neochlorogenic acid biosynthesis at 5 µM, whereas the highest isochlorogenic acid accumulation occurred in the presence of 5 µM BAP [8]. Additionally, 3 µM m-TOP enhanced flavonoid production in *Allamanda cathartica* cultures [54]. In *D. forrestii* shoots, BAP derivatives also promoted the formation of apigenin derivatives, although no clear relationship between cytokinin type or concentration and their levels was observed [22].

### 2.4. Antioxidant Activity of C. nepeta Culture Extract

The antioxidant potential of plant extracts is an important indicator of their medicinal value. As plant extracts represent complex mixtures of phytochemicals capable of undergoing diverse oxidative transformations, their antioxidant capacity is typically assessed using multiple complementary analytical assays. As such, in the present study, the antioxidant properties were analysed using three in vitro tests: DPPH, ABTS and FRAP assay.

**Figure 5:**
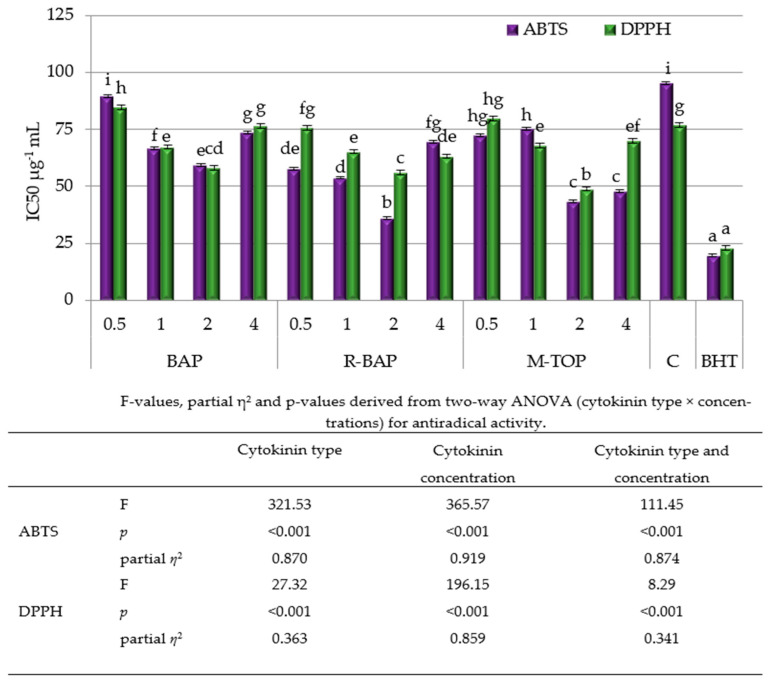
Free radical scavenging activity (DPPH and ABTS assays) of hydromethanolic extracts from shoots of *C. nepeta* grown on different concentrations of purine cytokinins. Results expressed as means of three replicates ± SE (calculated from a two-way ANOVA model). Means followed by at least one common letter are not significantly different according to Tukey’s HSD test (*p* ≤ 0.05), with “a” indicating the highest mean value.

The antioxidant activity of *C. nepeta* shoot extracts, expressed as IC_50_ (half maximal inhibitory concentration) in the antiradical ABTS and DPPH assays, was significantly influenced by cytokinin type and concentration, and their interaction (*p* < 0.001 for all factors; two-way ANOVA) (Figure 5).

In the radical scavenging assays, lower IC_50_ values corresponded to higher antioxidant activity. The strongest activity was observed for extracts derived from shoots treated with 2 mg L^−1^ m-TOP and r-BAP. In the DPPH assay, the lowest IC_50_ value was recorded for m-TOP at 2 mg L^−1^ (48.89 µg mL^−1^), followed by r-BAP at 2 mg L^−1^ (56.16 µg mL^−1^). A similar trend was observed in the ABTS assay. In contrast, the weakest antioxidant activity was observed in the control culture and for shoots cultivated on medium with 0.5 mg L^−1^ BAP (Figure 5).

**Figure 6:**
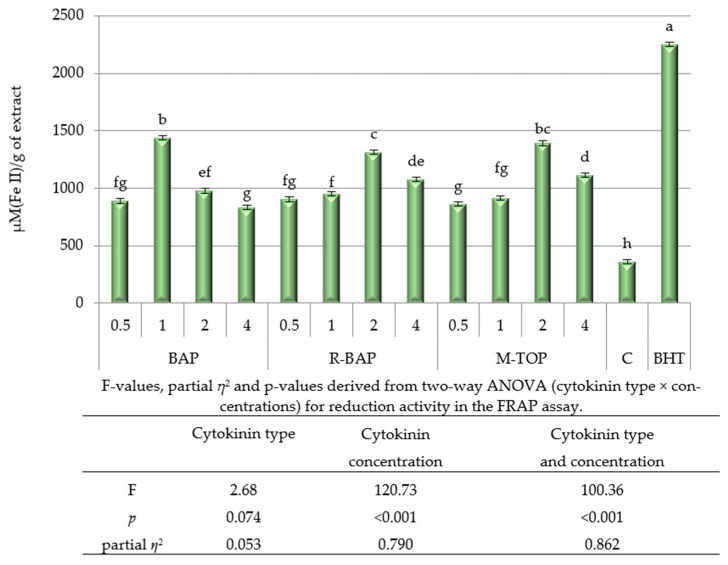
Ferrous ion chelating activity (FRAP) of hydromethanolic extracts from shoots of *C. nepeta* grown on different concentrations of purine cytokinins. Results expressed as means of three replicates ± SE (calculated from a two-way ANOVA model). Means followed by at least one common letter are not significantly different according to Tukey’s HSD test (*p* ≤ 0.05), with “a” indicating the highest mean value.

For the FRAP assay, extracts obtained from *C. nepeta* shoots cultured under different cytokinin treatments exhibited ferric reducing power values of approximately 832 to 1440 µmol Fe(II) g^−1^ DW, compared with 2253 µmol Fe(II) g^−1^ for the reference antioxidant BHT (Figure 6). The highest reducing capacity was observed for extracts obtained from cultures treated with 1 mg L^−1^ BAP followed by 2 mg L^−1^ m-TOP and 2 mg L^−1^ r-BAP, which is close to the patterns observed for radical scavenging activity.

The antioxidant activity of *C. nepeta* shoot extracts obtained in the present study should be interpreted in the context of both extract composition and processing. Compared with fractionated extracts, crude extracts typically exhibit lower activity due to the presence of non-active or interfering constituents. Accordingly, fractionated extracts of *C. nepeta*, particularly the butanol fraction enriched in phenolics, have been reported to show substantially stronger activity, with ABTS and DPPH IC_50_ values ranging from 9.56–21.04 µg mL^−1^ and 8.12–42.77 µg mL^−1^, respectively [12]. Nevertheless, the antioxidant activity observed herein is higher than that noted in non-fractionated extracts obtained from field-grown plants [10], suggesting that in vitro-derived biomass may constitute a more efficient source of antioxidant compounds, even without additional purification. This can be attributed to the controlled culture conditions, which enable targeted modulation of secondary metabolism and enhanced accumulation of bioactive metabolites.

The antioxidant properties of the analysed extracts appear to be largely associated with their phenolic composition. Metabolite profiling revealed high levels of phenolic constituents, including rosmarinic acid, chlorogenic acid derivatives, and flavonoid glycosides, predominantly acacetin derivatives, all of which are well-established contributors to antioxidant activity in Lamiaceae. Rather than operating via distinct mechanistic pathways, these polyphenolic classes share overlapping antioxidant mechanisms, including hydrogen atom transfer, single electron transfer, and transition metal chelation, with their relative contribution determined primarily by structural features influencing reaction kinetics and radical stabilization. Within this network of redox-active metabolites, both caffeoylquinic acids and flavonoids act as important, complementary contributors to the overall antioxidant capacity [55,56,57,58]. However, RA appears to play a dominant role due to its high abundance and exceptionally favourable structural configuration. As an ester of caffeic acid and 3,4-dihydroxyphenyllactic acid, it contains multiple ortho-dihydroxy systems, enabling highly efficient electron and hydrogen atom transfer reactions. This structural arrangement confers strong reactivity toward reactive oxygen and nitrogen species, while also facilitating transition metal chelation, thereby suppressing Fenton-type reactions. In addition, RA can modulate endogenous antioxidant defences, including superoxide dismutase, catalase, and glutathione-dependent systems, further amplifying its contribution to the overall antioxidant potential [59].

The observed relationship between phenolic composition and antioxidant activity is consistent with previous reports for *C. nepeta* and other Lamiaceae species [52,60]. Moreover, the results indicate that cytokinin-dependent modulation of phenolic metabolism may contribute to the observed changes in antioxidant activity, likely driven by coordinated shifts in quantitative composition of key metabolites. Similarly, elicitation with silver nanoparticles significantly increased RA accumulation (up to 4.5-fold), accompanied by enhanced antiradical activity and reduction potential of *C. nepeta* culture [52], further supporting the central role of this compound in determining bioactivity.

**Figure 7:**
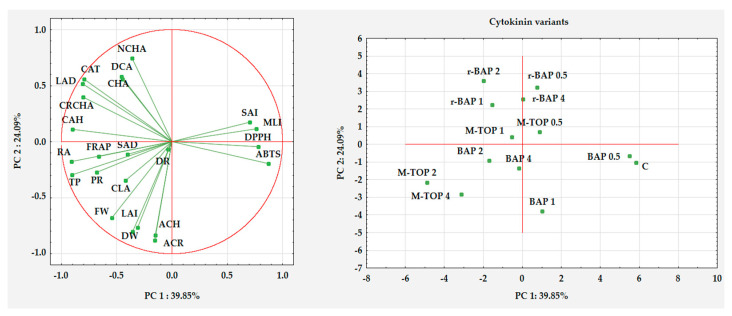
Principal component analysis (PCA) of growth parameters, phenolic metabolites, and antioxidant activity in *C. nepeta* shoot cultures cultivated on media supplemented with various cytokinins. The loading plot (**left**) illustrates the relationships among analysed variables: phenolic metabolites, total polyphenol content (TP), growth parameters (PR—proliferation rate, FW—fresh weight, DW—dry weight), and antioxidant activity determined by FRAP, ABTS, and DPPH assays (ABTS and DPPH expressed as IC_50_ values). The score plot (**right**) shows the distribution of cytokinin treatments (BAP, r-BAP, m-TOP at different concentrations) and the control (C). Abbreviations of metabolites: NCHA—neochlorogenic acid, DCA—dicaffeoyl glycoside, CAH—caffeoyl hexoside, CAT—caffeoyl-threonic acid derivative, CHA—chlorogenic acid, CRCHA—cryptochlorogenic acid, LAD—lithospermic acid derivative, RA—rosmarinic acid, CLA—clinopodic acid I, LAI—lithospermic acid isomer, SAD—salvianolic acid D derivative, SAI—salvianolic acid isomer, MLI—monomethyl lithospermate isomer, DR—diosmetin rutinoside, ACH—acetylhexosyl-rutinoside, ACR—acacetin 7-*O*-rutinoside.

To further elucidate the relationships among cytokinin treatments, biomass accumulation, phenolic metabolite profiles, and antioxidant capacity in *C. nepeta* shoot cultures, a principal component analysis (PCA) was performed (Figure 7).

Principal component analysis (PCA) explained 61.96% of the total variance, with PC1 and PC2 accounting for 39.85% and 24.09%, respectively (Figure 7). PC1 clearly separated variables associated with phenolic metabolite accumulation from antioxidant activity parameters expressed as IC_50_ values. Rosmarinic acid and most caffeoyl- and lithospermic acid derivatives (NCHA, CRCHA, CAT, CHA, LAD, DCA, CAH) were located on the negative side of PC1, whereas ABTS and DPPH IC_50_ values were positioned on the positive side. As antioxidant results are expressed as IC_50_, it can be seen that higher phenolic levels correspond to stronger antioxidant activity (i.e., lower IC_50_). The observed differences in antioxidant activity among cytokinin treatments appeared to coincide with quantitative changes in phenolic compounds, particularly with respect to rosmarinic acid accumulation. Growth-related traits (FW, DW and PR) were also associated with the negative PC1 region, suggesting a positive relationship between biomass production and several phenolic constituents.

PC2 further separated the metabolite groups, with most caffeoylquinic and lithospermic acid derivatives located in the upper part of the loading plot, and flavonoid glycosides, together with biomass parameters, positioned in the lower region. The cytokinin treatments were found to cluster corresponding to their effects on growth, phenolic accumulation, and antioxidant activity. Treatments such as higher m-TOP and, to a lesser extent, BAP 2 and r-BAP 1 and 2 were positioned on the negative side of PC1, indicating their association with higher biomass production and elevated levels of majority secondary metabolites, accompanied by generally stronger antioxidant activity (lower IC_50_ values). In contrast, the control (C) was located on the positive side of PC1, correlating with higher ABTS and DPPH IC_50_ values and thus weaker radical scavenging capacity.

Along PC2, treatments were further differentiated according to qualitative differences in metabolite profiles. Variants located in the upper region indicating a correlation with m-TOP 1 and moderate r-BAP contents were more closely associated with caffeoylquinic acid derivatives, whereas those in the lower part, indicating a stronger association with higher m-TOP variants, were linked to rosmarinic acid, flavonoid glycosides, acacetin derivatives, and biomass-related traits.

## 3. Materials and Methods

### 3.1. In Vitro Shoot Cultures

*Clinopodium nepeta* shoot cultures were established from seeds obtained from the Botanical Garden of the Medical University of Lublin (Poland). The seeds were surface-sterilized by immersion in 70% (*v*/*v*) ethanol for 15 s, followed by 1% (*v*/*v*) sodium hypochlorite for 5 min, and subsequently rinsed three times with sterile distilled water (15 min each). The sterilized seeds were inoculated onto Murashige and Skoog (MS) medium [61] solidified with 0.7% (*w*/*v*) agar to obtain aseptic seedlings.

Apical buds excised from five-week-old seedlings were transferred to hormone-free MS agar medium to initiate shoot cultures. Shoots were routinely subcultured at five-week intervals. Single nodal segments (~1 cm in length) excised from five-week-old shoots at the 5th–7th subculture were used as explants. The explants were cultured in culture tubes containing 25 mL of MS agar medium supplemented with indole-3-acetic acid (IAA; 0.2 mg L^−1^) and one of the following cytokinins: 6-benzylaminopurine (BAP), meta-topolin (m-Top), or riboside of BAP (r-BAP), applied at concentrations of 0.5, 1.0, 2.0 or 4 mg L^−1^. The cytokinin concentrations were selected based on previous reports concerning shoot proliferation in Lamiaceae species and on preliminary experiments; the aim was to cover the range expected to promote shoot multiplication while avoiding excessive physiological abnormalities [4,5,19]. Explants cultured on MS medium supplemented with IAA (0.2 mg L^−1^) alone served as controls. Shoot cultures were maintained in a growth chamber under a 16 h photoperiod with a light intensity of 50 μmol m^−2^ s^−1^ at 26 ± 2 °C.

The experiment was conducted in three independent biological replicates corresponding to three separate subcultures, each consisting of 20 explants per treatment. After five weeks of culture, the proliferation rate, expressed as the mean number of newly formed shoots per explant, shoot length (cm), and fresh and dry biomass (g per culture tube) were determined. Fresh weight (FW) was measured by weighing culture obtained from a single explant immediately after removal from the medium and careful elimination of agar residual. Subsequently, the plant material was lyophilized for 24 h to a constant weight and weighed again to determine dry weight (DW). Fresh and dry biomass were expressed as grams per culture tube. The frequency of hyperhydricity was assessed by visual evaluation of shoot morphology at the end of the culture period and expressed as the percentage of hyperhydric cultures relative to the total number of cultures evaluated within each treatment.

### 3.2. Chemicals

Growth medium and plant growth regulators used for the cultivation of *C. nepeta* shoots were purchased from Duchefa Biochemie (Haarlem, The Netherlands). All solvents used for chromatographic analyses (analytical grade) and reagents used for antioxidant assays were obtained from Sigma-Aldrich (Darmstadt, Germany). High-purity reference standards for quantitative analysis were used: chlorogenic acid, caffeic acid, and apigenin-7-*O*-glucoside were purchased from Sigma-Aldrich (Darmstadt, Germany), whereas rosmarinic acid and lithospermic acid were supplied by Chem Faces Biochemical Co., Ltd. (Wuhan, China).

### 3.3. Plant Material Extraction

Lyophilized shoots of *C. nepeta* (100 mg per sample) were finely pulverized and extracted three times with 20 mL of a methanol–water solution (8:2, *v*/*v*) for 15 min using an ultrasonic bath (UD-20 ultrasonic disintegrator; Techpan, Warsaw, Poland). The combined extracts were evaporated to dryness under reduced pressure, and the resulting crude extracts were subjected to phytochemical analysis.

### 3.4. Qualitative UHPLC–DAD–ESI–MS Analysis

Qualitative UHPLC–DAD–MS analyses were performed using an Ultimate 3000 UHPLC system (Dionex, Idstein, Germany) coupled via a splitless interface to an AmaZon SL ion trap mass spectrometer equipped with an electrospray ionization (ESI) source (Bruker Daltonik GmbH, Bremen, Germany). The mass spectrometer was operated under the following conditions: nebulizer pressure, 40 psi; dry gas flow rate, 9 L/min; dry gas temperature, 300 °C; capillary voltage, 4.5 kV. Mass spectra were acquired in the *m*/*z* range of 70–2200 in negative ionization mode. Tandem mass spectrometry (MS^2^) experiments were performed for the two most intense precursor ions. UV–Vis spectra were recorded in the range of 200–450 nm, and chromatograms were monitored at 254, 280, 325, and 350 nm. Chromatographic separation was achieved using a Kinetex XB-C18 column (150 mm × 2.1 mm, 1.7 μm particle size; Phenomenex, Torrance, CA, USA) maintained at 25 °C.

Prior to analysis, the samples were dissolved in 50% (*v*/*v*) methanol containing 0.1% (*v*/*v*) formic acid to obtain a final concentration of 10 mg/mL and filtered through a 0.45 μm PVDF syringe filter. Elution was carried out using a gradient system consisting of 0.1% (*v*/*v*) formic acid in water (solvent A) and 0.1% (*v*/*v*) formic acid in acetonitrile (solvent B) as follows: 5–17% B (0–15 min), 17–25% B (15–18 min), 25–60% B (18–36 min), and 60–99% B (36–45 min). The flow rate was set at 0.3 mL/min, and the injection volume was 5 μL.

### 3.5. Quantitative UHPLC Analysis

Quantitative analysis of polyphenolic compounds was performed using an Agilent Technologies 1290 Infinity UHPLC system (Santa Clara, CA, USA) equipped with a diode array detector (DAD). Chromatographic separation was achieved on an Eclipse XDB-C18 column (100 mm × 3.0 mm, 1.8 µm particle size; Agilent Technologies).

The mobile phase consisted of water (solvent A) and acetonitrile (solvent B), both acidified with formic acid (0.1%, *v*/*v*). Elution was carried out using the following gradient program: 5–17% B (0–9 min), 17–24% B (9–20 min), 24–59% B (20–25 min), 59–90% B (25–28 min), and 90–100% B (28–30 min). The flow rate was set at 0.6 mL/min, and the injection volume was 10 µL. The column temperature was maintained at 35 °C, and detection was performed at 325 nm.

Individual compounds were quantified based on external calibration curves. These were constructed using reference standards of analytical purity (>98%): chlorogenic acid (y = 2.4071x, R^2^ = 0.9995), rosmarinic acid (y = 1.9884x, R^2^ = 0.9995), caffeic acid (y = 6.2776x, R^2^ = 0.9995), lithospermic acid (y = 9.0868x, R^2^ = 0.9998), and apigenin-7-*O*-glucoside (y = 1.3576x, R^2^ = 0.9996). Compounds lacking authentic reference standards were quantified using calibration curves of structurally related compounds and expressed as equivalents of the corresponding reference standard: clinopodic acid, salvianolic acid D and a salvianolic acid isomer were quantified as rosmarinic acid equivalents; dicaffeoyl glycoside, caffeoyl hexoside and a caffeoyl-threonic acid derivative as caffeic acid equivalents; neochlorogenic acid and cryptochlorogenic acid as chlorogenic acid equivalents; a lithospermic acid derivative and a lithospermic acid isomer as lithospermic acid equivalents; diosmetin rutinoside, acacetin acetylhexosyl rutinoside, and acacetin-7-*O*-rutinoside as apigenin-7-O-glucoside equivalents. The results were expressed as mg g^−1^ dry weight (DW).

### 3.6. Antioxidant Assays

All antioxidant assays were performed using an Agilent BioTek Synergy H1 microplate reader (Agilent Technologies, Santa Clara, CA, USA) equipped for 96-well plate measurements and temperature control. Butylated hydroxytoluene (BHT) was used as a positive control.

#### 3.6.1. FRAP Microplate Assay

The ferric reducing antioxidant power (FRAP) assay was carried out using a microplate adaptation of the method described by Pulido et al. [62]. Briefly, 20 µL of the sample extract was mixed with 150 µL of freshly prepared FRAP reagent. After incubation, the absorbance was measured at 595 nm. A calibration curve was constructed using ferrous sulfate (FeSO_4_) standard solutions in the concentration range of 50–1500 µM. The results were expressed as µmol Fe(II) g^−1^ dry weight (DW) of extract.

#### 3.6.2. DPPH Radical Scavenging Assay

The free radical scavenging activity was evaluated using the DPPH (2,2-diphenyl-1-picrylhydrazyl) assay according to Grzegorczyk-Karolak and Kiss [60]. The extracts were tested at concentrations ranging from 5 to 500 µg mL^−1^. Aliquots of 100 µL of each sample were mixed with 100 µL of a methanolic DPPH solution (0.2 mM) in a 96-well microplate. The reaction mixtures were incubated in the dark at room temperature for 30 min, and the absorbance was measured at 517 nm. Antiradical activity was expressed as IC_50_ values (µg mL^−1^), defined as the concentration of the extract required to scavenge 50% of DPPH radicals.

#### 3.6.3. ABTS Radical Scavenging Assay

The ABTS [2,2′-azino-bis(3-ethylbenzothiazoline-6-sulfonic acid)] radical scavenging assay was performed according to Re et al. [63]. The extracts were prepared at concentrations ranging from 5 to 500 µg mL^−1^. For the assay, 100 µL of ABTS•^+^ working solution was mixed with 100 µL of the sample in a 96-well microplate and incubated at room temperature for 10 min. Absorbance was then measured at 734 nm. The results were expressed as IC_50_ values (µg mL^−1^), corresponding to the concentration of the extract required to scavenge 50% of ABTS radicals.

### 3.7. Statistical Analysis

All results were expressed as the mean ± standard error (SE) of three independent experimental biological replicates (subcultures). For phytochemical and antioxidant analyses, one sample was prepared from each biological replicate (*n* = 3), and each sample was analysed in triplicate as technical replicates. Preliminary data processing was performed using Microsoft Excel (Microsoft Corporation, Redmond, WA, USA). Statistical significance was evaluated by two-way analysis of variance (ANOVA), followed by Tukey’s HSD post hoc test, with differences considered significant at *p* ≤ 0.05. Principal component analysis was performed to evaluate the relationships between cytokinin treatments, growth parameters, phenolic metabolite accumulation, and antioxidant activity in the *C. nepeta* shoot cultures. The analysis included quantitative data for individual metabolites, total polyphenol content, proliferation rate (PR), fresh weight (FW), dry weight (DW) and antioxidant activity determined using FRAP, ABTS and DPPH assays. Prior to analysis, the dataset was autoscaled (mean-centred and standardized to unit variance) to eliminate the influence of different measurement units. PCA was performed using the covariance matrix, and the results were visualized as loading and score plots representing the first two principal components (PC1 and PC2). These components explained the majority of variance in the dataset and were used to identify correlations among variables and clustering patterns among cytokinin treatments. All statistical analyses were conducted using Statistica software version 13.1 PL (StatSoft Inc., Kraków, Poland).

## 4. Conclusions

This study provides the first comprehensive evaluation of the effects of selected cytokinins on shoot morphogenesis, biomass accumulation, phenolic metabolism, and antioxidant activity in *Clinopodium nepeta* in vitro cultures. Both cytokinin type and concentration significantly affected growth and metabolite production. Among the tested growth regulators, meta-topolin proved the most effective, promoting high multiplication rates, favourable shoot morphology, enhanced biomass accumulation, and elevated production of phenolic compounds, particularly rosmarinic acid, whose accumulation exceeded 23 mg g^−1^ DW and was nearly twenty-fold higher than in control cultures. UHPLC–DAD–ESI–MS profiling confirmed the presence of a diverse spectrum of phenolic metabolites characteristic of the Lamiaceae family. Cytokinin-treated cultures generally exhibited enhanced antioxidant activity, accompanied by treatment-related changes in phenolic metabolite levels. These findings indicate that *C. nepeta* shoot cultures represent a promising system for the controlled production of phenolic-rich biomass and provide a useful platform for investigating cytokinin-dependent modulation of secondary metabolism.

While the present study provides valuable insights into cytokinin-mediated regulation of growth and phenolic metabolism in *C. nepeta*, several limitations should be considered. The study was conducted using a single shoot culture line derived from one accession, some metabolites were quantified using structurally related surrogate standards, and no molecular analyses were performed to elucidate the mechanisms underlying cytokinin-induced responses. Future research should focus on validating these findings across additional genotypes, elucidating the molecular regulation of phenolic biosynthesis, and further optimizing metabolite production through elicitation approaches and bioreactor-based cultivation systems.

## Figures and Tables

**Table 1:** Effect of cytokinins on *C. nepeta* culture growth.

	**Shoot** **Formation (%)**	**Propagation Rate**	**Shoot Length (cm)**	**Hyperhydricity** **(%)**
BAP 0.5	70 ± 10.0	1.81 ± 0.11 ^f^	1.86 ± 0.88 ^b^	0 ± 0.0
BAP 1	70 ± 12.1	2.61 ± 0.09 ^cde^	1.40 ± 0.06 ^cd^	0 ± 0.0
BAP 2	83.3 ± 15.0	2.00 ± 0.19 ^ef^	1.45 ± 0.06 ^c^	10 ± 2
BAP 4	83 ± 12.3	4.00 ± 0.20 ^a^	0.95 ± 0.05 ^d^	25 ± 3.8
r-BAP 0.5	85 ± 8.2	2.13 ± 0.08 ^ef^	1.84 ± 0.11 ^b^	0 ± 0.0
r-BAP 1	82 ± 7.1	2.29 ± 0.12 ^def^	1.57 ± 0.09 ^bc^	0 ± 0.0
r-BAP 2	82 ± 8.0	2.84 ± 0.14 ^cd^	1.30 ± 0.07 ^cd^	5 ± 0.5
r-BAP 4	80.5 ± 5.6	3.22 ± 0.16 ^bc^	1.25 ± 0.06 ^cd^	11 ± 1.5
m-TOP 0.5	55 ± 3.6	2.23 ± 0.12 ^def^	1.76 ± 0.09 ^b^	0 ± 0.0
m-TOP 1	70 ± 6.4	2.64 ± 0.14 ^cde^	1.65 ± 0.08 ^bc^	0 ± 0.0
m-TOP 2	81.4 ± 9.2	3.61 ± 0.16 ^ab^	1.50 ± 0.07 ^bc^	3 ± 0.5
m-TOP 4	83 ± 6.0	4.48 ± 0.21 ^a^	1.25 ± 0.07 ^cd^	3 ± 0.2
control	61 ± 4.1	1.35 ± 0.08 ^g^	2.65 ± 0.35 ^a^	0 ± 0.0
F-values, partial *η*^2^ and *p*-values derived from two-way ANOVA (cytokinin type × concentrations) for growth parameters.				
		**Cytokinin Type**	**Cytokinin** **Concentration**	**Cytokinin Type** **and Concentration**
	F	23.79	84.541	9.426
Propagation rate	*p*	<0.001	<0.001	<0.001
	partial *η*^2^	0.117	0.413	0.136
	F	2.60	39.19	2.50
Shoot length	*p*	0.075	<0.001	0.022
	partial *η*^2^	0.014	0.246	0.040

Results expressed as means of three replicates ± SE (calculated from a two-way ANOVA model). Means followed by at least one common letter are not significantly different according to Tukey’s HSD test (*p* ≤ 0.05), with “a” indicating the highest mean value.

**Table 2:** Secondary metabolites detected in hydromethanolic extract of *C. nepeta* by UPLC-DAD/ESI-MS/MS.

Peak No.	Tentative Compound	Rt [min]	UV-Vis [nm]	[M − H]^−^ *m*/*z*	MS^2^ Ions	References
1	neochlorogenic acid (NCHA)	7.1	212, 295 sh, 325	353	191 b, 179, 135	[20,34,35]
2	dicaffeoyl glycoside (DCA)	7.9	212, 288 sh, 324	503	179	[36]
3	caffeoyl hexoside (CAH)	8.9	216, 295 sh, 324	341	221	[36]
4	caffeoyl-threonic acid isomer (CAT)	9.2	216, 297 sh, 326	297	297	[36,37]
5	chlorogenic acid (CHA)	10.9	216, 293 sh, 324	353	191	[20,35]
6	lithospermic acid derivative (LAD)	11.2	216, 274	711	537 b, 493, 295	[38]
7	cryptochlorogenic acid (CRCHA)	11.8	216, 294, 325	353	191, 173 b	[34,35]
8	diosmetin rutinoside (DR)	22.3	218, 267, 332	607	443, 299 b, 284	[36,39]
9	salvianolic acid D derivative (SAD)	22.6	218, 289, 316	553	373, 355 b, 329, 311	[40]
10	rosmarinic acid (RA)	23.0	198, 216, 286, 328	359	223, 161 b	[33,41]
11	clinopodic acid I (CLA)	23.8	218, 287, 329	717	555, 519 b, 475, 357	[41]
12	lithospermic acid isomer (LAI)	24.0	218, 289 sh, 323	537	493 b, 359, 313	[38]
13	acacetin acetylhexosyl-rutinoside [+HCOO^−^] (ACH)	24.4	217, 268, 330	841	795 b, 753, 736, 283	[42,43]
14	acacetin 7-*O*-rutinoside [+HCOO^−^] (ACR)	24.7	217, 267, 330	637	591, 283 b, 268	[41,44]
15	salvianolic acid isomer (SAI)	25.4	219, 286, 329	537	519 b, 493, 439, 357	[36]
16	monomethyl lithospermate isomer (MLI)	26.6	220, 287, 325	551	519 b, 359, 339, 223, 161	[45]

b—base peak (the most abundant ion in the recorded spectrum); sh—shoulder in UV–Vis spectrum; +HCOO^−^—adduct from formic acid.

**Table 3:** The effect of purine cytokinins on accumulation of caffeic acid monomers and dimers in *C. nepeta* culture.

Compounds (mg g^−1^ DW)						
Cytokinin (mg/L)	NCHA	DCA	CAH	CAT	CHA	CRCHA
BAP 0.5	0.129 ± 0.001 ^h^	0.122 ± 0.001 ^d^	0.034 ± 0.006 ^g^	0.032 ± 0.0002 ^i^	0.107 ± 0.001 ^g^	0.248 ± 0.003 ^i^
BAP 1	0.074 ± 0.002 ^j^	0.049 ± 0.001 ^i^	0.078 ± 0.0003 ^f^	0.027 ± 0.001 ^i^	0.117 ± 0.002 ^g^	0.286 ± 0.002 ^h^
BAP 2	0.159 ± 0.001 ^d^	0.171 ± 0.001 ^a^	0.100 ± 0.002 ^e^	0.305 ± 0.003 ^g^	0.346 ± 0.007 ^b^	0.895 ± 0.004 ^f^
BAP 4	0.155 ± 0.001 ^e^	0.114 ± 0.001 ^e^	0.070 ± 0.001 ^f^	0.278 ± 0.002 ^i^	0.332 ± 0.002 ^b^	0.842 ± 0.03 ^g^
r-BAP 0.5	0.182 ± 0.002 ^b^	0.166 ± 0.001 ^bc^	0.108 ± 0.002 ^d^	0.415 ± 0.001 ^d^	0.393 ± 0.007 ^b^	1.085 ± 0.009 ^c^
r-BAP 1	0.195 ± 0.001 ^a^	0.166 ± 0.001 ^b^	0.117 ± 0.001 ^cd^	0.423 ± 0.001 ^c^	0.392 ± 0.002 ^a^	1.081 ± 0.006 ^c^
r-BAP 2	0.173 ± 0.001 ^c^	0.163 ±0.001 ^bc^	0.114 ± 0.001 ^cd^	0.525 ± 0.001 ^a^	0.294 ± 0.002 ^d^	1.022 ± 0.003 ^d^
r-BAP 4	0.144 ± 0.001 ^g^	0.106 ± 0.001 ^f^	0.079 ± 0.001 ^f^	0.475 ± 0.001 ^b^	0.211 ± 0.001 ^e^	0.973 ± 0.001 ^e^
m-TOP 0.5	0.146 ± 0.001 ^fg^	0.103± 0.001 ^f^	0.116 ± 0.003 ^bc^	0.305 ± 0.001 ^g^	0.313 ± 0.002 ^c^	0.972 ± 0.005 ^e^
m-TOP 1	0.143 ± 0.001 ^g^	0.110 ± 0.001 ^e^	0.125 ± 0.001 ^b^	0.374 ± 0.001 ^f^	0.295 ± 0.002 ^d^	0.975 ± 0.004 ^e^
m-TOP 2	0.149 ± 0.001 ^ef^	0.162 ± 0.001 ^c^	0.169 ± 0.002 ^a^	0.479 ± 0.001 ^b^	0.222 ± 0.003 ^e^	1.216 ± 0.004 ^a^
m-TOP 4	0.129± 0.004 ^h^	0.089 ± 0.001 ^g^	0.121 ± 0.002 ^bc^	0.402 ± 0.002 ^e^	0.178 ± 0.004 ^f^	1.154 ± 0.013 ^b^
control	0.122 ± 0.001 ^i^	0.076 ± 0.001 ^h^	0.019 ± 0.003 ^h^	0.040 ± 0.003 ^h^	0.109 ± 0.001 ^g^	0.109 ± 0.001 ^j^
F-values, partial *η*^2^ and *p*-values derived from two-way ANOVA (cytokinin type × concentrations) for studied metabolite levels.						
Cytokinin type						
F	1794.2	2523.0	818.79	57,373.5	765.6	9708.6
*p*	<0.001	<0.001	<0.001	<0.001	<0.001	<0.001
partial *η*^2^	0.974	0.981	0.945	0.999	0.941	0.995
Cytokinin concentration						
F	292.0	3680.3	227.90	13,767.2	86.78	1803.0
*p*	<0.001	<0.001	<0.001	<0.001	<0.001	<0.001
partial *η*^2^	0.901	0.991	0.877	0.998	0.731	0.983
Cytokinin type and concentration						
F	777.5	1515.9	60.81	2142.5	1059.13	1185.4
*p*	<0.001	<0.001	<0.001	<0.001	<0.001	<0.001
partial *η*^2^	0.979	0.989	0.792	0.993	0.985	0.987

Results expressed as means of three replicates ± SE (calculated from a two-way ANOVA model). Means followed by at least one common letter are not significantly different according to Tukey’s HSD test (*p* ≤ 0.05), with “a” indicating the highest mean value. Neochlorogenic acid—NCHA, dicaffeoyl glycoside—DCA, caffeoyl hexoside—CAH, caffeoyl-threonic acid isomer—CAT, chlorogenic acid—CHA, cryptochlorogenic acid—CRCHA.

**Table 4:** The effect of purine cytokinins on accumulation of trimers and tetramers of caffeic acid in *C. nepeta* culture.

Compounds (mg g^−1^ DW)						
Cytokinin (mg/L)	LAD	SAD	LAI	CLA	SAI	MLI
BAP 0.5	0.038 ± 0.0002 ^k^	0.303 ± 0.001 ^g^	0.415 ± 0.004 ^f^	0.358 ± 0.001 ^j^	0.159 ± 0.001 ^b^	0.146 ± 0.001 ^b^
BAP 1	0.072 ± 0.002 ^j^	0.532 ± 0.014 ^a^	0.563 ±0.003 ^d^	0.769 ± 0.001 ^e^	-	-
BAP 2	0.391 ± 0.002 ^f^	0.427 ± 0.003 ^e^	0.802 ± 0.003 ^b^	1.517 ± 0.003 ^b^	-	-
BAP 4	0.297 ± 0.0003 ^i^	0.266 ± 0.003 ^h^	0.875 ± 0.002 ^a^	1.580 ± 0.003 ^a^	-	-
r-BAP 0.5	0.493 ± 0.001 ^d^	0.434 ± 0.0004 ^e^	0.306 ± 0.003 ^g^	0.646 ± 0.003 ^i^	0.179 ± 0.002 ^a^	0.148 ± 0.002 ^b^
r-BAP 1	0.446 ± 0.001 ^e^	0.415 ± 0.003 ^e^	0.285± 0.001 ^h^	0.672 ± 0.002 ^g^	-	-
r-BAP 2	0.547 ± 0.001 ^a^	0.502 ± 0.002 ^b^	0.199 ± 0.002 ^k^	0.712 ± 0.002 ^f^	-	-
r-BAP 4	0.524 ± 0.0002 ^b^	0.472 ± 0.001 ^c^	0.187 ± 0.002 ^l^	0.739 ± 0.003 ^f^	-	-
m-TOP 0.5	0.349 ± 0.0004 ^h^	0.309 ± 0.006 ^g^	0.221 ± 0.002 ^j^	0.659 ± 0.002 ^h^	-	-
m-TOP 1	0.382 ± 0.001 ^g^	0.342 ± 0.001 ^f^	0.239 ± 0.001 ^i^	0.733 ± 0.003 ^f^	-	-
m-TOP 2	0.488 ± 0.001 ^d^	0.524 ± 0.006 ^ab^	0.780 ± 0.005 ^c^	0.912 ± 0.002 ^d^	-	-
m-TOP 4	0.505 ± 0.002 ^c^	0.503 ± 0.002 ^b^	0.802 ± 0.003 ^b^	0.985 ± 0.004 ^c^	-	-
control	0.454 ± 0.001 ^e^	0.450 ± 0.007 ^d^	0.447 ± 0.001 ^e^	0.723 ± 0.005 ^f^	0.129 ± 0.013 ^c^	0.158 ± 0.008 ^a^
F-values, partial *η*^2^ and *p*-values derived from two-way ANOVA (cytokinin type × concentrations) for studied metabolite levels.						
Cytokinin type						
F	97,694	222.52	25,670.8	23,510	6602.03	6241.70
*p*	<0.001	<0.001	<0.001	<0.001	<0.001	<0.001
partial *η*^2^	0.999	0.823	0.999	0.998	0.993	0.992
Cytokinin concentration						
F	26,141	383.49	10,596.1	35,502	26,124.61	24,962.45
*p*	<0.001	<0.001	<0.001	<0.001	<0.001	<0.001
partial *η*^2^	0.999	0.923	0.997	0.999	0.999	0.999
Cytokinin type and concentration						
F	5132	389.20	5675.7	14,882	6602.03	6241.70
*p*	<0.001	<0.001	<0.001	<0.001	<0.001	<0.001
partial *η*^2^	0.997	0.961	0.997	0.999	0.998	0.997

Results expressed as means of three replicates ± SE (calculated from a two-way ANOVA model). Means followed by at least one common letter are not significantly different according to Tukey’s HSD test (*p* ≤ 0.05), with “a” indicating the highest mean value. Lithospermic acid derivative—LAD, salvianolic acid D derivative—SAD, clinopodic acid I—CLA, lithospermic acid isomer—LAI, salvianolic acid isomer—SAI, monomethyl lithospermate isomer—MLI. (-)—content below the quantification limit.

**Table 5:** The effect of purine cytokinins on flavonoids accumulation in *C. nepeta* culture.

Compounds (mg g^−1^ DW)			
Cytokinin (mg/L)	DR	ACH	ACR
BAP 0.5	0.295 ± 0.001 ^h^	3.076 ± 0.021 ^e^	3.116 ± 0.034 ^c^
BAP 1	0.308 ± 0.001 ^h^	3.483 ± 0.015 ^c^	3.483 ± 0.015 ^b^
BAP 2	0.348 ± 0.005 ^g^	3.615 ± 0.006 ^b^	3.445 ± 0.047 ^b^
BAP 4	0.306 ± 0.001 ^h^	3.228 ± 0.003 ^d^	3.043 ± 0.003 ^c^
r-BAP 0.5	0.485 ± 0.003 ^e^	2.814 ± 0.015 ^f^	2.611 ± 0.012 ^ef^
r-BAP 1	0.448 ± 0.002 ^f^	2.609 ± 0.037 ^g^	2.544 ± 0.027 ^f^
r-BAP 2	0.340 ± 0.002 ^g^	1.695 ± 0.003 ^i^	1.744 ± 0.002 ^g^
r-BAP 4	0.331 ± 0.001 ^g^	1.513 ± 0.005 ^j^	1.785 ± 0.003 ^g^
m-TOP 0.5	0.940 ± 0.009 ^a^	2.505 ± 0.022 ^h^	2.509 ± 0.008 ^f^
m-TOP 1	0.729 ± 0.001 ^b^	2.803 ± 0.004 ^f^	2.706 ± 0.019 ^e^
m-TOP 2	0.676 ± 0.007 ^c^	3.777 ± 0.025 ^a^	3.660 ± 0.054 ^a^
m-TOP 4	0.605 ± 0.004 ^d^	3.668 ± 0.003 ^b^	3.686 ± 0.008 ^a^
control	0.722 ± 0.004 ^b^	2.912 ± 0.037 ^g^	2.805 ± 0.04 ^d^
F-values, partial *η*^2^ and *p*-values derived from two-way ANOVA (cytokinin type × concentrations) for studied flavonoid level.			
Cytokinin type			
F	13,116.7	5812.6	2209.4
*p*	<0.001	<0.001	<0.001
partial *η*^2^	0.996	0.992	0.979
Cytokinin concentration			
F	911.6	141.0	37.0
*p*	<0.001	<0.001	<0.001
partial *η*^2^	0.966	0.815	0.536
Cytokinin type and concentration			
F	443.6	1453.8	483.3
*p*	<0.001	<0.001	<0.001
partial *η*^2^	0.965	0.989	0.968

Results expressed as means of three replicates ± SE (calculated from a two-way ANOVA model). Means followed by at least one common letter are not significantly different according to Tukey’s HSD test (*p* ≤ 0.05), with “a” indicating the highest mean value. Diosmetin rutinoside—DR, acacetin acetylhexosyl-rutinoside—ACH, acacetin 7-O-rutinoside—ACR.

## Data Availability

The original contributions presented in this study are included in the article. Further inquiries can be directed to the corresponding author(s).
